# Point-of-Care Diagnostic Biosensors to Monitor Anti-SARS-CoV-2 Neutralizing IgG/sIgA Antibodies and Antioxidant Activity in Saliva

**DOI:** 10.3390/bios13020167

**Published:** 2023-01-20

**Authors:** Eiichi Tamiya, Shuto Osaki, Tomoko Tsuchihashi, Hiromi Ushijima, Keiichi Tsukinoki

**Affiliations:** 1 Advanced Photonics and Biosensing Open Innovation Laboratory, National Institute of Advanced Industrial Science and Technology, Photonics Center, Osaka University, 2-1 Yamadaoka, Suita 565-0871, Osaka, Japan; 2 SANKEN, Osaka University, 8-1 Mihogaoka, Ibaraki 567-0047, Osaka, Japan; 3 BioDevice Technology Ltd., 2-3 Asahidai, Nomi 923-1211, Ishikawa, Japan; 4 Department of Environmental Pathology, Kanagawa Dental University, 82 Inaoka-cho, Yokosuka 238-0003, Kanagawa, Japan

**Keywords:** salivary biomarkers, gold-linked electrochemical immunosensor, electrochemiluminescence, anti-SARS-CoV-2 neutralizing antibodies, antioxidant activity, point-of-care diagnosis

## Abstract

Monitoring biomarkers is a great way to assess daily physical condition, and using saliva instead of blood samples is more advantageous as the process is simple and allows individuals to test themselves. In the present study, we analyzed the titers of neutralizing antibodies, IgG and secretory IgA (sIgA), in response to the SARS-CoV-2 vaccine, in saliva. A total of 19 saliva and serum samples were collected over a 10-month period 3 weeks after the first vaccine, 8 months after the second vaccine, and 1 month after the third vaccine. The ranges of antibody concentrations post-vaccination were: serum IgG: 81–15,000 U/mL, salivary IgG: 3.4–330 U/mL, and salivary IgA: 58–870 ng/mL. A sharp increase in salivary IgG levels was observed after the second vaccination. sIgA levels also showed an increasing trend. A correlation with trends in serum IgG levels was observed, indicating the possibility of using saliva to routinely assess vaccine efficacy. The electrochemical immunosensor assay developed in this study based on the gold-linked electrochemical immunoassay, and the antioxidant activity measurement based on luminol electrochemiluminescence (ECL), can be performed using portable devices, which would prove useful for individual-based diagnosis using saliva samples.

## 1. Introduction

Early diagnosis in the field and at home can contribute to disease detection, the prevention of severe diseases, and the control of infection. While physical and blood tests are used to diagnose diseases, the use of saliva instead of blood would facilitate the screening of a large number of individuals as well as providing diagnoses in dispersed areas, such as point-of-care (POC) and home-based testing. In particular, saliva collection does not require a medical professional, as is the case in blood sampling, and saliva self-collection can enable routine medical services in remote areas without reliance on urban clinics and hospitals. There are two main types of antibodies detected in saliva: secretory IgA (sIgA) and IgG. sIgA is produced locally in the salivary glands, while most IgG in saliva is transferred into saliva through the gingival crevices after being produced in the plasma cells. SARS-CoV-2 is transmitted to humans via the oral and nasal cavities, causing severe acute respiratory syndrome. The squamous epithelium of the tongue and periodontal tissue of the gums contain epithelial cells that express angiotensin-converting enzyme-2 (ACE-2), the receptor for SARS-CoV-2. Saliva also contains substances such as lactoferrin, lysozyme, and most abundantly, sIgA, which inhibit infection. We have identified SARS-CoV-2 cross-reactive IgA spike proteins in the saliva of people without COVID-19, suggesting that sIgA may inhibit the binding of ACE-2 to spike proteins. This reveals the importance of sIgA as a suppressor of SARS-CoV-2 infection in the oral cavity [1,2].

In addition, the IgG and sIgA spike proteins against SARS-CoV-2 (full-length trimer) and their receptor-binding domains (RBDs), found in the serum and saliva of COVID-19 patients in the acute and convalescent phases, positively correlate with saliva samples and may be surrogates for systemic immunity against SARS-CoV-2, indicating that their presence may serve as potential indicators of infection [3]. Correlations were also examined using blood and saliva samples from individuals with different histories, such as healthy (no vaccine), pre-infected, and vaccinated (once and twice) individuals. The results indicated that saliva samples may be able to monitor the decline in immune response following vaccination, although they were slightly less sensitive and specific than blood samples [4]. The salivary antibody levels of positive individuals were significantly higher than those of negative individuals and correlated well with antibody levels in the serum and plasma [5]. Asymptomatic infected individuals have higher sIgA levels than symptomatic infected individuals, suggesting an anti-disease protective role of sIgA antibodies [5]. Unvaccinated children showed evidence of exposure almost exclusively through specific sIgA responses despite the absence of evidence of viral infection [6]. An increase in sIgA levels has also been observed after the onset of COVID-19 [7]. Thus, monitoring salivary antibody responses following infection and vaccination can provide useful information regarding vaccination strategies. Therefore, POC immunosensors are essential for easy monitoring of more individuals on a daily basis.

Saliva contains not only antibody molecules such as sIgA and IgG for defense against infection, but also various antioxidants as prophylactics. Superoxide dismutase (SOD), catalase, glutathione, and peroxidase are preventive antioxidants that inhibit the formation of reactive oxygen species (ROS). Vitamins A and E, uric acid (UA), and bilirubin are responsible for clearing ROS and contain the spread of cell damage [8]. Inflammation caused by microbial and viral infections in the oral cavity is known to activate leukocytes, produce ROS, and reduce the antioxidant activity of saliva [9]. It is also known to be correlated with periodontal disease and dental caries [9,10]. ROS generated in the body changes the oxidative state of cells and predisposes an individual to various diseases [8], suggesting that knowledge of the antioxidant status of saliva can be an indicator of an individual’s health. Saliva can be collected noninvasively and used as a diagnostic fluid to detect biomarkers of various pathological conditions [11]. To evaluate the biomarkers, electrochemical/electrochemiluminescence (ECL) biosensors have attained much research interest because of their portability, low cost, stability, high sensitivity, and easy handling [12,13,14,15].

Therefore, portable electrochemical/ECL biosensors for detecting salivary biomarkers are gaining attention. The detection principle of biosensors differs according to the biomarker and has been developed for hormones [16,17], ions [18,19], glucose [20], cancer protein markers, pathogens [21], and SARS-COV-2 antibodies [13,22,23,24,25,26,27,28].

**Scheme 1:**
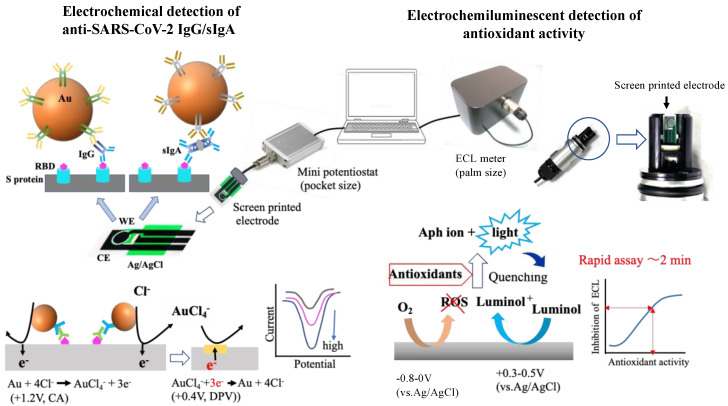
This scheme indicates the electrochemical detection of anti−SARS−CoV−2 IgG and IgA (**left part**) and the electrochemiluminescent detection of antioxidant activity (**right part**). Both electrochemical and luminescent measuring devices were capable of POC monitoring with disposable, mass-producible printed electrodes. The principle underlying the measurements is explained in the text.

The platform used in this study for the measurement of IgA, IgG, and antioxidants is shown in Scheme 1. To measure neutralizing active antibodies against SARS-CoV-2, the RBD-S1 protein was immobilized on an electrode. IgG and sIgA antibodies specific to the S protein in saliva were bound to the electrode, and the antigen–antibody reaction was quantified using gold nanoparticles modified with anti-human IgA and anti-human IgG antibodies. Gold nanoparticles were used on the electrode to measure the current caused by their ionization through an oxidation and a subsequent reduction reaction with high sensitivity, using the differential pulse voltammetry (DPV) method, whereby the potential is applied in a pulsed manner, and the difference between the current values before and after the pulse is output; thus, the contribution of the charging current is small, and the Faraday current can be measured efficiently. The reaction equation for gold nanoparticles is Au + Cl_4_^−^ ⇄ AuCl_4_ + 3e^−^ ($E_{0}$ = 0.803 V, vs. Ag/AgCl sat.), and the chloride ions not only act as electrolytes but also decrease the standard redox potential ($E_{0}$) of gold. The potential of oxidation to oxidize gold nanoparticles is still high, and it is effective at oxidizing all the gold nanoparticles before measuring the reduction current. Therefore, the measurement was performed using the reduction current as an indicator and examining its correlation with the amount of antibody.

This method was invented by our group and is called the gold-linked electrochemical immunoassay (GLEIA), which enables the measurement of gonadotropin [29], C-reactive protein [30], and sIgA [31,32]. This method utilizes the electrochemical activity and antibody-modifying function of gold nanoparticles and has been shown to function effectively as an immunosensor on a scale of 10–100 nm. Based on this principle, high sensitivity has been achieved using printed electrodes modified with carbon nanotubes (CNTs) [33] and graphene [34].

On the other hand, to measure antioxidant activity, as shown in the scheme below (right), an electrode reaction was used to generate reactive oxygen species from dissolved oxygen that would then react with oxidized luminol to form high-energy intermediates by eventually forming ions emitting at 420 nm. Any molecule with antioxidant activity consumes the ROS produced on the electrode, thus quenching the luminescence from luminol. Nagatani et al. reported that the amount of quenched luminescence correlated with the antioxidant activity, and that the antioxidant activity of food can be measured rapidly (in approximately 2 min) [35]. Whereas other methods require the addition of an external reagent to generate oxygen-active species, this method allows for the generation of oxygen-active species from dissolved oxygen simply by applying an electrical potential. In addition, luminol luminescence can be induced by oxygen-active species simply by changing the potential on the same electrode. In a previous study, we created a calibration curve for trolox, a standard for antioxidant capacity, measured the luminescence intensity in 22 different types of drinking water, and calculated the antioxidant capacity from the calibration curve. The obtained results were compared with the antioxidant capacity obtained via ORAC (Oxygen Radical Absorbance Capacity), and a high correlation was observed. A correlation was also observed for the scavenging capacity for superoxide radicals and hydroxyl radicals by ESR [35]. A pocket-sized electrochemical measuring device and palm-sized electrochemiluminescence device were used and operated under PC control. In addition, printed electrodes, which are mass-producible, inexpensive, and disposable, were used to ensure safety from biological human samples, including infectious samples.

In this study, an electrochemical biosensor system was developed for the rapid detection of neutralizing antibodies (sIgA and IgG) against SARS-CoV-2 and antioxidant activity in saliva samples, which can easily be collected daily. The results obtained were evaluated to demonstrate the usefulness of the biosensor for POC diagnosis.

## 2. Materials and Methods

### 2.1. Reagents, S Proteins, and Antibodies

Gold nanoparticles with a 60 nm diameter (EMGC60) were purchased from BBI Solutions (Cardiff, UK). Three different recombinants—the S1-mFc recombinant protein (40591-V05H1, Sino Biological, Beijing, China), the S1 + S2 recombinant with His-tag (BSV-CoV-PR-40, BioServ, San Diego, CA, USA), and the RBD recombinant protein with an Fc tag (BSV-CoV-PR-09, BioServ) were purchased. As positive controls for neutralizing antibodies, the sIgA antibody (E-AB-V1027, Elabscience, Houston, TX, USA), IgG antibody (SPD-M180, Acro Biosystems, Tokyo, Japan), and IgG standard in the IgG ELISA kit (290-84201, FUJIFILM Wako, Osaka, Japan) were used. Anti-human IgA (A80-102A) and anti-human IgG (2049-01) were obtained from Bethyl Laboratories (Waltham, MA, USA) and Southern Biotech (Birmingham, AL, USA), respectively. Bovine serum albumin (BSA) was purchased from Sigma-Aldrich (St. Louis, MO, USA). Polyethylene glycol 20,000 (PEG) was obtained from Fluka. Luminol, trolox, ascorbate, trehalose dihydrate, and Bradford protein assay kits were purchased from FUJIFILM Wako.

### 2.2. Electrodes and Instruments

A pocket-sized potentiostat (miniSTAT100) and a palm-sized electrochemiluminometer (BDTeCL-XP) were obtained from BioDevice Technology (Ishikawa, Japan). Both pictures are shown in Scheme 1. Screen-printed electrodes (EP-P, EP-PP) with an integrated working electrode (2.64 mm^2^ diameter), counter electrode, and Ag/AgCl reference electrode, with a total length of 11 mm, were also obtained from BioDevice Technology (Ishikawa, Japan). Two UV-visible spectrometers, U-2900 (Hitachi, Japan) and DS-11 (DeNovix, Wilmington, DE, USA), were used for the quantification of gold nanoparticles and proteins, respectively. A micro-high-speed cooling centrifuge (KUBOTA3700, Kubota, Tokyo, Japan) was also used in this study for the preparation of antibody-coated gold nanoparticles.

### 2.3. Preparation of Secondary Antibody-Coated Gold Nanoparticles

Anti-human IgA and anti-human IgG antibodies were used as secondary antibodies. Secondary antibody-modified gold nanoparticles were prepared using our previously reported method [31]. The antibodies (50 μg/mL) were dissolved in 5 mM phosphate buffer (pH 7.5), added to the Au nanoparticle solution, and then, incubated for 10 min at room temperature. This is known as the Au conjugate, which was mixed with 0.1 mL of 10% BSA in phosphate buffer and 0.05 mL of 1% PEG (0.1 mL) in phosphate buffer. Au and anti-IgA/anti-IgG conjugates were collected via centrifugation (8000× *g* for 15 min at 4 °C). Then, the Au anti-IgA/anti-IgG conjugate was suspended in 1 mL of preservation solution (1% BSA, 0.05% PEG, 0.1% NaN_3_, and 150 mM NaCl in 20 mM Tris-HCl buffer, pH 8.2) and collected again in the same manner. For the stock solution, the Au anti-IgA/anti-IgG conjugate was suspended in the preservation solution and the optical density was adjusted to OD_520_ = 6. The Au anti-IgA/anti-IgG conjugate was diluted three times with trehalose (OD_520_ = 2), and 3 µL of this solution was added to each well of the 96-well plate. The plate was then dried under vacuum for 5 min. In this case, 60 nm diameter gold nanoparticles were selected, because they showed a better response than the 20 nm and 100 nm AuNPs from our previous study [32].

### 2.4. Immobilization of S Protein Antigens on the Working Electrode

The S protein (2 µL) in phosphate buffer (50 µg/mL) was dropped onto the working electrode and incubated at room temperature for 1 h to adsorb the protein. The amount of antibody adsorbed on the electrode was evaluated via electrochemical impedance spectroscopy (EIS) and 50 ug/mL was determined to be adequate for this study (see Figure S3).

Then, 10 µL of 1% BSA in phosphate buffer was dropped onto the entire electrode, and this was incubated at room temperature for 1 h to suppress non-specific adsorption.

### 2.5. Sandwich-Type Neutralizing Antibody and Gold Nanoparticles Modified with Anti-IgA/IgG on the Working Electrode and Electrochemical Detection of Gold Nanoparticles

A sandwich-type reaction occured directly on the working electrode, as shown in Scheme 1. IgA/IgG test solutions (10 µL) were added onto the 96-well plate containing Au anti-IgA/anti-IgG conjugates, and mixed for 10 s. Then, 1.4 µL of the solution was placed on the working electrode and incubated for 15 min at room temperature for IgG detection. After rinsing with a phosphate buffer, the solution was removed. The direct redox reaction was performed in a 0.5M HCl solution (30 µL) covering the entire electrode at room temperature. The pre-oxidation of Au nanoparticles was performed at a constant potential of 1.2 V for 40 s, immediately followed by DPV, while scanning the potential range from 0.6 to 0.1 V with a step potential of 4.0 mV, a pulse amplitude of 50 mV, and a pulse period of 0.2 s. The DPV conditions were determined according to our previous report [29,30,31]. The potentials were recorded against the Ag/AgCl electrode.

### 2.6. ECL Measurement for Determination of Antioxidant Activity

The antioxidant activity of the saliva samples was determined by diluting them 5-fold and mixing them with a luminol solution (final concentration: 100 µM, pH 8.1). The potential window of CV was kept between −800 mV and +800 mV, and the other CV operating parameters included a scan rate of 50 mV/s and a time interval of 0.1 s. The trigger signal was sent to the photon detection unit from the potentiostat at the same time as the electrochemical measurement was started, and the ECL intensity was measured every 0.1 s. Antioxidant standard curves were prepared using different concentrations of ascorbate and trolox.

### 2.7. Protein Concentration

Protein concentrations were determined using Bradford assay reagent with BSA as the standard control. The measuring instrument used was a DeNovix DS 11 with a wavelength of 595 nm. Saliva samples were diluted 10–20 times as required and mixed with Bradford reagent, and 3 µL was used for the measurements.

### 2.8. Saliva Collection from Selected Participants

We collected samples using Salivettes1 (Sarstedt AG & Co., KG, Numbrecht, Germany) in a hospital room between 9 a.m. and 12 p.m. The participants were instructed to refrain from eating, drinking, and brushing their teeth for at least 1 h before sample collection. Saliva samples were immediately centrifuged at 2000× *g* for 15 min, and then, stored at –80 °C. We tested the samples for SARS-CoV-2 using polymerase chain reaction (PCR). Individuals with saliva samples were confirmed to be negative for COVID-19 upon PCR testing in the study. The participants were volunteers from Kanagawa Dental University. Individuals with IgA nephropathy, selective IgA deficiency, autoimmune diseases, or cold-like symptoms within the past 2 weeks were excluded from the study. This study was approved by the Kanagawa Dental University Research Ethics Review Board (approval number: 792). This study was registered in the Japanese UMIN Clinical Trials Registry (UMIN-CTR) (approval numbers: #UMIN000047028 and UMIN000043717), and therefore, meets the ICMJE standards.

## 3. Results and Discussion

### 3.1. Choice of Antigens and Calibration for Neutralizing IgG and sIgA Antibodies

To measure antibodies that show neutralizing activity, we chose viral S proteins that reacted selectively with neutralizing antibodies on the electrode surface. Spike proteins are transmembrane proteins that contain two subunits: S1 and S2. S1 mainly contains the receptor-binding domain (RBD) and is responsible for recognizing cell surface angiotensin receptors, while S2 contains the basic elements required for membrane fusion. Three recombinant S proteins were tested in this study: the S1-mFc recombinant protein, the S1 + S2 recombinant with His-tag, and the RBD recombinant protein with Fc tag, which correspond to amino acid sequences 16–685, 16–1210, and 319–541 of the S protein, respectively. The results showed that the S1 + S2 region recombinant protein had good calibration properties for both IgG and IgA. Although the RBD region is the most useful binding site for assessing neutralizing activity, as a sensor, it showed a high background signal, even at low concentrations. This could be due to partial changes in the protein structure upon immobilization on the electrode surface. The neutralizing activity of various types of antibodies against SARS-CoV-2 in serum has been reported [36] and the results have shown that the RBD, S1 domain, full-length S protein, and S trimer have high specificity (RBD: 98%, S1: 97%, S full: 93%, and S trimer: 96%). In terms of sensitivity, the full-length S protein had greater sensitivity than the RBD to a large extent, and the S trimer had greater sensitivity than the S1 domain, but to a smaller extent.

**Figure 1:**
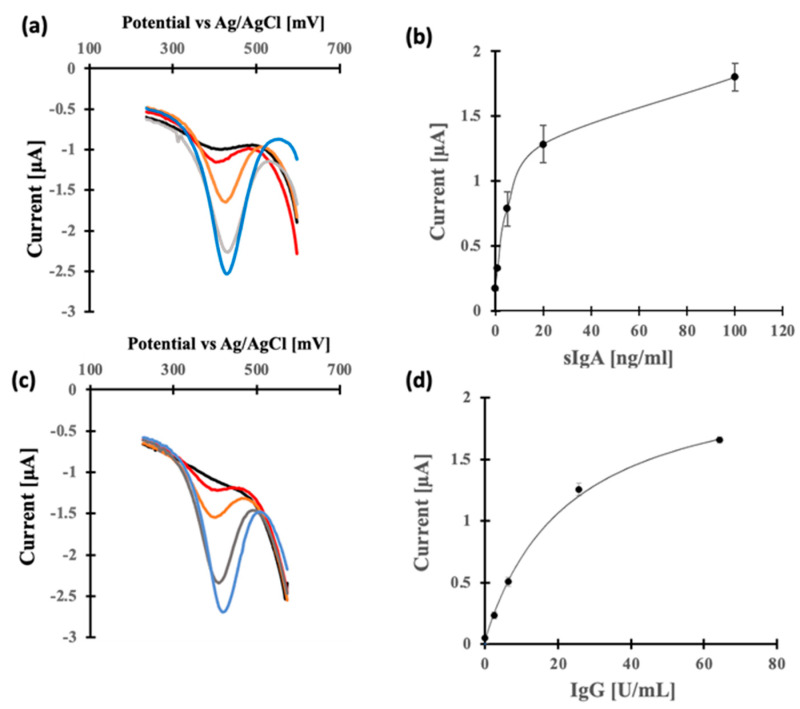
Current-potential profiles (**a**,**c**) and calibration curves (**b**,**d**) of neutralizing antibodies sIgA and IgG were obtained using the GLEIA−based electrochemical immunosensor. Current−potential responses were tested under different concentrations of (**a**) sIgA 100 ng/mL (blue), 20 ng/mL (grey), 5 ng/mL (orange), 1 ng/mL (red), and 0 ng/mL (black) and (**c**) IgG 62.5 U/mL (blue), 25 U/mL (grey), 6.25 U/mL (orange), 2.5 U/mL (red), and 0 U/mL (black). Calibration curves were indicated for sIgA (**b**) and IgG (**d**) as Michaelis–Menten-type functions by a non-linear curve fitted in the graphing software Origin2022 (OriginLab).

The electrochemical immunosensor responses and calibration curves for the neutralizing antibodies sIgA and IgG are shown in Figure 1. Calibrations were obtained as Michaelis–Menten type functions via non-linear curve fitting in graphing software. The detection limit was 0.28 U/mL for IgG. This value was determined using three-sigma limits. The range of both assays was almost two orders of magnitude; therefore, the assayed sample had to be diluted. The ELISA kit had a measurement range of 2–250 U/mL of neutralizing antibody activity, indicating that our GLEIA system was more sensitive. The ELISA measurement required 3 h, including repeated washing and enzymatic reaction operations, and was not suitable as POC diagnostic equipment. In addition, saliva samples contain high concentrations of enzyme proteins and mucins (in the order of 100 µg/mL), which needed to be diluted to reduce their effect on the antigen–antibody reaction. The GLEIA system showed a high background signal without dilution (Figure S1 in Supplementary Materials). This was attributed to the non-specific adsorption of antibody-modified gold nanoparticles on the electrode. Blocking conditions have been extensively studied during the development of GLEIA. In the present study, a 100-fold dilution was used because a 10-fold dilution showed a lower correlation coefficient with the ELISA method (less than 0.4).

### 3.2. Antioxidant Activity of Saliva Samples

**Figure 2:**
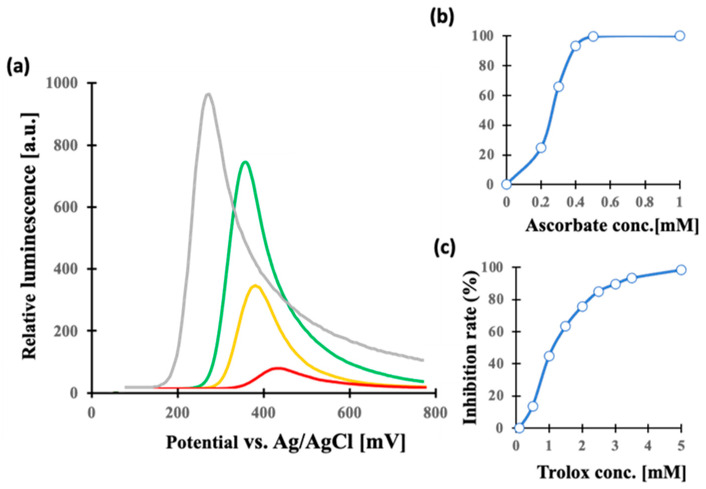
Antioxidant activity was determined using luminol electrochemiluminescence as described in the text. Luminescence-potential profiles (**a**) were obtained with different concentrations of ascorbic acid: 0 mM (grey), 0.2 mM (green), 0.3 mM (yellow), and 0.4 mM (red). Luminescence inhibition rate (%) was calculated using the formula: [1 − (luminescence at zero concentration − luminescence of sample)/(luminescence at zero concentration)] × 100 (%). Luminescence inhibition rates are plotted against the concentrations of ascorbic acid (**b**) and trolox (**c**).

Luminol electrochemiluminescence was used to measure the antioxidant activity of saliva. This method, reported by us, uses the luminescence produced by oxidized luminol at +0.3–0.4 V (vs. Ag/AgCl) with the reactive oxygen species produced via the reduction of dissolved oxygen at −0.8–0.5 V (vs. Ag/AgCl). Antioxidant molecules inhibit ROS production and quench luminescence. Different beverage products were tested using trolox as a standard for measuring the antioxidant activity in food [24]. The results presented here are based on ascorbic acid and trolox as a representative of antioxidant substances (Figure 2).

Antioxidant activity was plotted as the rate of luminescence inhibition (%), using the formula: [1 − (luminescence at zero concentration − luminescence with sample)/(luminescence at zero concentration)] × 100 (%)

The rate of luminescence inhibition corresponded to antioxidant activity in concentration ranges of 0.2–0.5 mM and 0.5–5 mM for ascorbic acid and trolox, respectively.

### 3.3. Applications for Monitoring Human Saliva Samples

**Figure 3:**
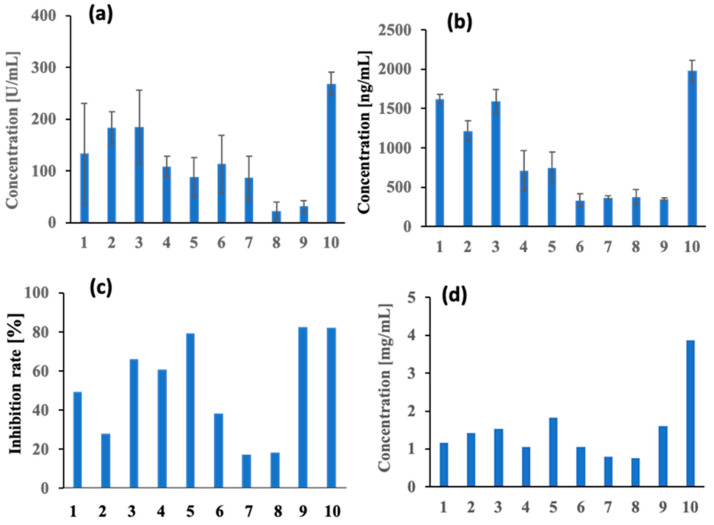
Comparisons of concentrations of neutralizing antibody IgG (**a**) and sIgA (**b**), antioxidant activity (indicated by luminescence inhibition rate) (**c**), and protein concentration (**d**) using saliva samples from 10 individuals are presented together. All 10 saliva samples were collected 3 weeks after the second dose of the vaccine.

Human saliva samples were collected from 10 individuals without COVID-19 infection. All 10 saliva samples were collected 3 weeks after the second dose of the vaccine. The saliva samples were tested for the neutralizing antibodies IgG and sIgA, antioxidant activity, and protein concentration (Figure 3). Each saliva sample was diluted 100-fold to suppress the effect of non-specific adsorption while measuring neutralizing antibodies. Salivary IgG concentrations were 10–100 times lower than their concentration in serum and were close to the detection limit for samples. However, the concentrations were below the detection limit for the ELISA kit. Individual variations in sIgA were observed at 0.334–1.97 µg/mL, which was within the range of variation reported for uninfected saliva samples [7]. As the total amount of sIgA in saliva is 65–145 µg/mL, it was also shown that only approximately 0.5–1% of sIgA has neutralizing activity. sIgA is a useful diagnostic indicator of an individual’s health, due to its affinity for various pathogens as mucosal antibodies. Therefore, routine pre-onset identification of sIgA based on individual characteristics may enable early detection to prevent the onset of disease. In contrast, there was a variation of 29 ± 25–268 ± 22 U/mL (almost the same as ng/mL) for IgG-neutralizing antibodies. Because the total amount of IgG in saliva is 20–30 µg/mL [37], the neutralizing antibody corresponds to 0.1–1%. This value is similar to that of sIgA.

**Figure 4:**
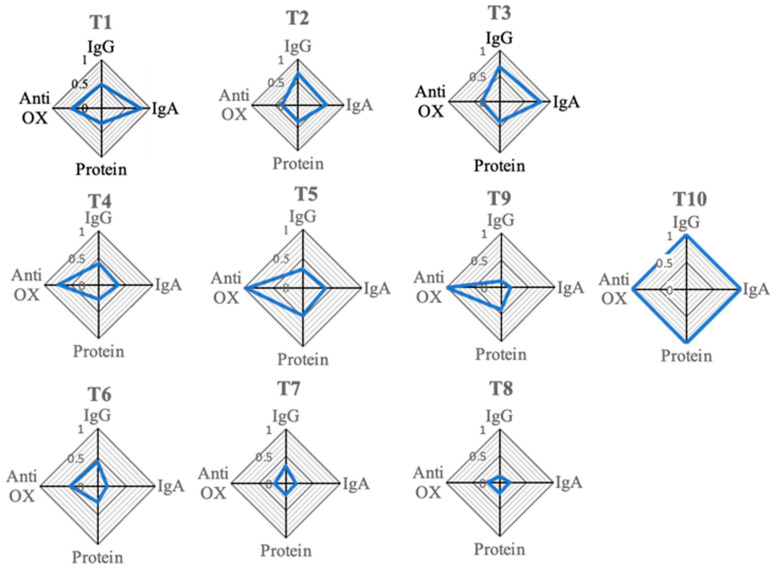
The four datasets of neutralizing antibodies—IgG and sIgA, antioxidant activity, and protein concentration—measured in 10 samples are depicted individually in a radar chart for each individual. The numbers T1–T10 correspond to the sample numbers 1–10 in Figure 3. The data are shown as a ratio of the maximum concentration of each measured item.

Antioxidant activity was determined using 5–10-fold dilutions, and the antibody activity was measured relative to the suppression of luminescence in the test vs. the control sample. The results showed individual variability of 17.3–82.4 (relative activity). The antioxidant activity of salivary components is made up of enzyme molecules that inhibit the formation of ROS, such as SOD, catalase, and peroxidase, as well as those that bind to and remove radical species, such as vitamins A and E, uric acid (UA), and glutathione. Inflammation caused by microbial and viral infections in the oral cavity activates leukocytes, which generate reactive oxygen species and reduce the antioxidant activity of saliva [9,10]. It has been suggested that knowing the antioxidant status of saliva can be an indicator of an individual’s health status. There are few reports on the changes in salivary antioxidant activity before and after the onset of COVID-19, and it would be useful to accumulate data on an individual basis. Protein levels varied between 0.76 and 3.83 mg/mL (BSA was used as standard). Sample 10 showed a high value of 3.83 mg/mL, while samples 1–9 were in the range of 0.76–1.82 mg/mL. In saliva, enzymes such as amylase (0.48 mg/mL), lysozyme (0.01 mg/mL), peroxidase (0.06 mg/mL), and the polysaccharide mucin (0.2 mg/mL) were present in high concentrations [38]. The four datasets of neutralizing activity—IgG and sIgA concentrations, antioxidant activity, and the protein concentration of the saliva samples from 10 individuals (T1–T10)—are shown in a radar chart for each individual (Figure 4). The data are presented as the ratio of the maximum concentration of each measured item. Samples 1–3 showed relatively high sIgA and IgG concentrations and relatively low antioxidant activity and protein concentration. Samples 6–8 showed a low-value pattern for all items. These results were considered to be due to the individual characteristics and conditions at the time of sampling. Daily data accumulation may be useful for individual health diagnoses.

### 3.4. Monitoring Changes in Saliva Markers over Time, after Multiple Vaccinations

**Figure 5:**
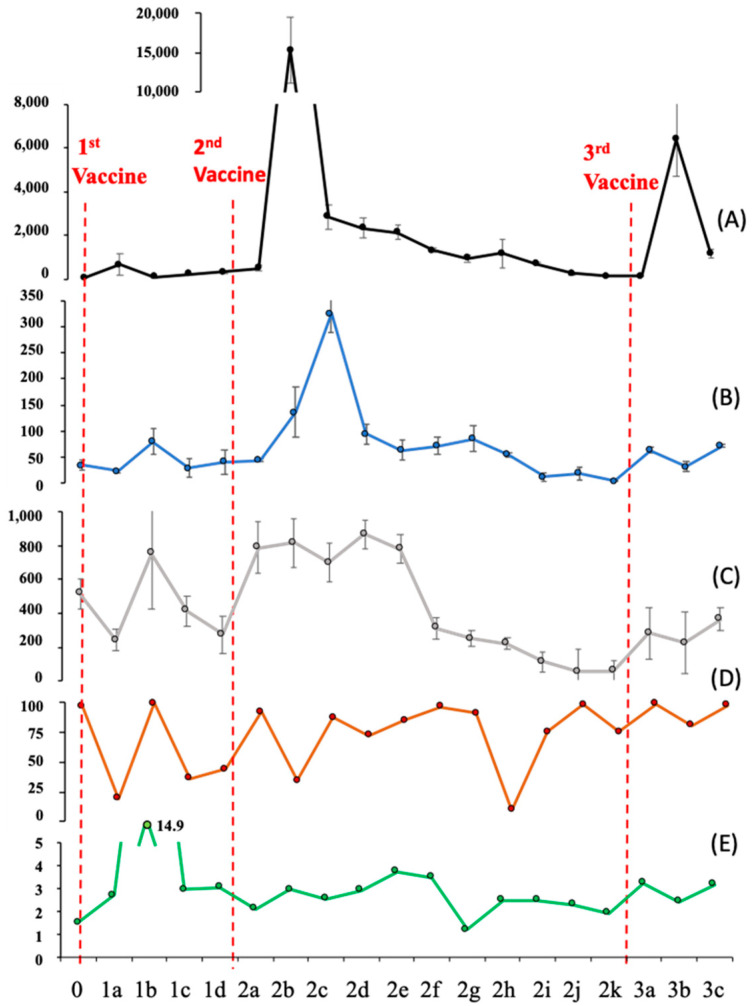
The results of continuous monitoring of the concentration of neutralizing IgG (**B**) and sIgA (**C**) antibodies, antioxidant activity (**D**), and protein concentration (**E**) in saliva and neutralizing IgG concentration in serum (**A**) before and after 1st, 2nd, and 3rd vaccinations in the same individual over time were compared vertically. The abscissa represents the date and time of sampling. 0: at first vaccination; 1a: 4 days later, 1b: 1 week later, 1c: 2 weeks later, and 1d: 3 weeks later; 2a: 4 days after second vaccination; 2b: 1 week later, 2c: 2 weeks later, 2d: 3 weeks later, 2e: 1 month later, 2f: 2 months later, 2g: 3 months later, 2h: 4 months later, 2i: 5 months later, 2j: 6 months later, and 2k: 8 months later; 3a: 3 days after the third vaccination, 3b: 2 weeks later, and 3c: 1 month later. The units on the vertical axis are as follows: (**A**,**B**) U/mL, (**C**) ng/mL, (**D**) %, and (**E**) mg/mL.

We measured IgA, IgG, antioxidant activity, and protein concentration in the saliva of an individual without COVID-19 infection (without periodontal disease or subjective symptoms of oral inflammation or bleeding before and after three vaccinations over time) (Figure 5 and Figure S2). Simultaneously, IgG measurements in the serum were performed and compared. Nineteen saliva and serum samples were collected over a 10-month period 3 weeks after the first vaccine, 8 months after the second vaccine, and 1 month after the third vaccine. The range of data measured during the study period was as follows: serum IgG: 81 U/mL (0)–15,000 U/mL(2b); salivary IgG: 3.4 U/mL (2k)–330 U/mL (2d); and sIgA: 58 ng/mL (2k)–870 ng/mL (2c). The serum IgG level was 660 U/mL 4 days after the first vaccination. IgG in the serum increased significantly to 15,000 U/mL at 1 week after the second vaccination, clearly indicating the effect of the vaccine. The salivary IgG levels increased from 33 U/mL on the day of the first vaccination to 79 U/mL 1 week later. It also increased from 140 U/mL 1 week after the second vaccination to 330 U/mL 2 weeks later. Interestingly, the peak was observed later than that for serum IgG. Following the third vaccination, an increasing trend in IgG was observed in both serum and saliva samples, although the increase was not as large as that after the second vaccination. These results indicate that vaccine efficacy can be monitored using saliva samples, without the need for blood sampling. As for sIgA, the concentration increased to 980 ng/mL after the second vaccination, which was approximately double the pre-vaccination level; after the third vaccination, it increased to 280 ng/mL, which was lower than that in the second vaccination, but significantly higher than the pre-vaccination level. sIgA is secreted into saliva through the salivary glands, and IgG is secreted into saliva through periodontal fluid; however, the dynamics of their presence in saliva after vaccination are not understood. The accumulation of such data over time, together with antioxidant activity and protein concentrations, could be useful as a basis for diagnosis and care based on individual characteristics.

## 4. Conclusions

In this study, we analyzed salivary titers of neutralizing antibodies against the SARS-CoV-2 vaccine, IgG and secreted IgA (sIgA), using a portable electrochemical biosensor. Thorough the continuous monitoring of saliva and serum samples from 19 subjects for 10 months, the concentration ranges of the antibodies were: serum IgG: 81–15,000 U/mL, salivary IgG: 3.4–330 U/mL, and salivary sIgA: 58–870 ng/mL. After the second vaccination, a sharp increase in serum IgG occurred, and salivary IgG and sIgA tended to follow. The tracking of saliva-neutralizing antibodies provided the possibility to determine the efficacy of the vaccine.

We also analyzed salivary antioxidant activity using a portable ECL biosensor. The results included lifestyle and individual differences among the subjects, and therefore, there is room for additional research. Daily data accumulation may be useful for individual health diagnoses.

To evaluate the utility of electrochemical biosensors, post-vaccination salivary biomarkers were monitored. Our GLEIA method and ECL biosensor show sufficient utility for on-site salivary biomarker evaluation. In the future, we will work to develop a more practical POC biosensor by expanding the number of subjects and the types of biomarkers to be evaluated.

## Data Availability

The data that support the findings of this study are available from the corresponding authors upon reasonable request.

## Supplementary Materials

The following supporting information can be downloaded at: https://www.mdpi.com/article/10.3390/bios13020167/s1, Figure S1: Optimization of the dilution rate for saliva sample; Figure S2: Preliminary check of blood contamination on saliva samples and Figure S3: Optimization of primary antibody concentration using the electrochemical impedance spectroscopy (EIS).

## References

[1] Tsukinoki K., Yamamoto T., Handa K., Iwamiya M., Saruta J., Ino S., Sakurai T. (2021). Detection of cross-reactive immunoglobulin A against the severe acute respiratory syndrome-coronavirus-2 spike 1 subunit in saliva. PLoS ONE.

[2] Tsukinoki K., Yamamoto T., Saito J., Sakaguchi W., Iguchi K., Inoue Y., Ishii S., Sato C., Yokoyama M., Shiraishi Y. (2022). Prevalence of saliva immunoglobulin A antibodies reactive with severe acute respiratory syndrome coronavirus 2 among Japanese people unexposed to the virus. Microbiol. Immunol..

[3] Isho B., Abe K.T., Zuo M., Jamal A.J., Rathod B., Wang J.H., Li Z., Chao G., Rojas O.L., Bang Y.M. (2020). Persistence of serum and saliva antibody responses to SARS-CoV-2 spike antigens in COVID-19 patients. Sci. Immunol..

[4] Lahdentausta L., Kivimaki A., Oksanen L., Tallgren M., Oksanen S., Sanmark E., Salminen A., Geneid A., Sairanen M., Paju S. (2022). Blood and saliva SARS-CoV-2 antibody levels in self-collected dried spot samples. Med. Microbiol. Immunol..

[5] Dobano C., Alonso S., Vidal M., Jimenez A., Rubio R., Santano R., Barrios D., Pons Tomas G., Mele Casas M., Hernandez Garcia M. (2022). Multiplex Antibody Analysis of IgM, IgA and IgG to SARS-CoV-2 in Saliva and Serum from Infected Children and Their Close Contacts. Front. Immunol..

[6] Thomas A.C., Oliver E., Baum H.E., Gupta K., Shelley K.L., Long A.E., Jones H.E., Smith J., Hitchings B., Bartolo N.D. (2022). Evaluation of isotype specific salivary antibody assays for detecting previous SARS-CoV-2 infection in children and adults. medRxiv.

[7] Varadhachary A., Chatterjee D., Garza J., Garr R.P., Foley C., Letkeman A.F., Dean J., Haug D., Breeze J., Traylor R. (2020). Salivary anti-SARS-CoV-2 IgA as an accessible biomarker of mucosal immunity against COVID-19. medRxiv.

[8] Minic I. (2019). Antioxidant Role of Saliva. J. Otolaryngol. Res..

[9] Tartaglia G.M., Gagliano N., Zarbin L., Tolomeo G., Sforza C. (2017). Antioxidant capacity of human saliva and periodontal screening assessment in healthy adults. Arch. Oral Biol..

[10] Komatsu T., Kobayashi K., Morimoto Y., Helmerhorst E., Oppenheim F., Chang-il Lee M. (2020). Direct evaluation of the antioxidant properties of salivary proline-rich proteins. J. Clin. Biochem. Nutr..

[11] Mohammed H.K., Anjana G., Zareena M.A., Sunil E.A. (2017). Antioxidant Capacity of Saliva: Effect on Onset and Progression of Dental Caries. Oral Maxillofac. Pathol. J..

[12] Huang Y., Yao Y., Wang Y., Chen L., Zeng Y., Li L., Guo L. (2022). Strategies for Enhancing the Sensitivity of Electrochemiluminescence Biosensors. Biosensors.

[13] Deng X., Lin X., Zhou H., Liu J., Tang H. (2023). Equipment of Vertically-Ordered Mesoporous Silica Film on Electrochemically Pretreated Three-Dimensional Graphene Electrodes for Sensitive Detection of Methidazine in Urine. Nanomaterials.

[14] Umapathi R., Ghoreishian S.M., Sonwal S., Rani G.M., Huh Y.S. (2022). Portable electrochemical sensing methodologies for on-site detection of pesticide residues in fruits and vegetables. Coord. Chem. Rev..

[15] Gong J., Tang H., Wang M., Lin X., Wang K., Liu J. (2022). Novel three-dimensional graphene nanomesh prepared by facile electro-etching for improved electroanalytical performance for small biomolecules. Mater. Des..

[16] Huang Z., Chen H., Ye H., Chen Z., Jaffrezic-Renault N., Guo Z. (2021). An ultrasensitive aptamer-antibody sandwich cortisol sensor for the noninvasive monitoring of stress state. Biosens. Bioelectron..

[17] Klinghammer S., Voitsekhivska T., Licciardello N., Kim K., Baek C.K., Cho H., Wolter K.J., Kirschbaum C., Baraban L., Cuniberti G. (2020). Nanosensor-Based Real-Time Monitoring of Stress Biomarkers in Human Saliva Using a Portable Measurement System. ACS Sens..

[18] Osaki S., Kintoki T., Moriuchi-Kawakami T., Kitamura K., Wakida S.I. (2019). Investigation of Polyurethane Matrix Membranes for Salivary Nitrate ISFETs to Prevent the Drift. Sensors.

[19] Lim H.R., Lee S.M., Mahmood M., Kwon S., Kim Y.S., Lee Y., Yeo W.H. (2021). Development of Flexible Ion-Selective Electrodes for Saliva Sodium Detection. Sensors.

[20] Garcia-Carmona L., Martin A., Sempionatto J.R., Moreto J.R., Gonzalez M.C., Wang J., Escarpa A. (2019). Pacifier Biosensor: Toward Noninvasive Saliva Biomarker Monitoring. Anal. Chem..

[21] Campuzano S., Yánez-Sedeño P., Pingarrón J.M. (2017). Electrochemical bioaffinity sensors for salivary biomarkers detection. TrAC Trends Anal. Chem..

[22] Abid S.A., Ahmed Muneer A., Al-Kadmy I.M.S., Sattar A.A., Beshbishy A.M., Batiha G.E., Hetta H.F. (2021). Biosensors as a future diagnostic approach for COVID-19. Life Sci..

[23] Rashed M.Z., Kopechek J.A., Priddy M.C., Hamorsky K.T., Palmer K.E., Mittal N., Valdez J., Flynn J., Williams S.J. (2021). Rapid detection of SARS-CoV-2 antibodies using electrochemical impedance-based detector. Biosens. Bioelectron..

[24] Gong J., Zhang T., Luo T., Luo X., Yan F., Tang W., Liu J. (2022). Bipolar silica nanochannel array confined electrochemiluminescence for ultrasensitive detection of SARS-CoV-2 antibody. Biosens. Bioelectron..

[25] Kumar N., Shetti N.P., Jagannath S., Aminabhavi T.M. (2022). Electrochemical sensors for the detection of SARS-CoV-2 virus. Chem. Eng. J..

[26] Kumar S., Patel A., Lai L., Chakravarthy C., Valanparambil R., Reddy E.S., Gottimukkala K., Davis-Gardner M.E., Edara V.V., Linderman S. (2022). Structural insights for neutralization of Omicron variants BA.1, BA.2, BA.4, and BA.5 by a broadly neutralizing SARS-CoV-2 antibody. Sci. Adv..

[27] Li F.F., Liu A., Gibbs E., Tanunliong G., Marquez A.C., Gantt S., Frykman H., Krajden M., Morshed M., Prystajecky N.A. (2022). A novel multiplex electrochemiluminescent immunoassay for detection and quantification of anti-SARS-CoV-2 IgG and anti-seasonal endemic human coronavirus IgG. J. Clin. Virol..

[28] Sadique M.A., Yadav S., Khare V., Khan R., Tripathi G.K., Khare P.S. (2022). Functionalized Titanium Dioxide Nanoparticle-Based Electrochemical Immunosensor for Detection of SARS-CoV-2 Antibody. Diagnostics.

[29] Idegami K., Chikae M., Kerman K., Nagatani N., Yuhi T., Endo T., Tamiya E. (2008). Gold Nanoparticle-Based Redox Signal Enhancement for Sensitive Detection of Human Chorionic Gonadotropin Hormone. Electroanalysis.

[30] Gondoh-Noda Y., Kometani M., Nomura A., Aono D., Karashima S., Ushijima H., Tamiya E., Murayama T., Yoneda T. (2020). Feasibility of a Novel Mobile C-Reactive Protein-Testing Device Using Gold-Linked Electrochemical Immunoassay: Clinical Performance Study. JMIR Mhealth Uhealth.

[31] Osaki S., Wakida S.I., Saito M., Tamiya E. (2021). Towards On-site Determination of Secretory IgA in Artificial Saliva with Gold-Linked Electrochemical Immunoassay (GLEIA) Using Portable Potentiostat and Disposable Printed Electrode. Appl. Biochem. Biotechnol..

[32] Osaki S., Espulgar W.V., Wakida S.-i., Saito M., Tamiya E. (2022). Optimization of electrochemical analysis for signal amplification in gold nanoparticle-probed immunoassays. Electrochim. Acta.

[33] Xuan Viet N., Chikae M., Ukita Y., Maehashi K., Matsumoto K., Tamiya E., Hung Viet P., Takamura Y. (2013). Gold-linked electrochemical immunoassay on single-walled carbon nanotube for highly sensitive detection of human chorionic gonadotropin hormone. Biosens. Bioelectron..

[34] Lim S.A., Yoshikawa H., Tamiya E., Yasin H.M., Ahmed M.U. (2014). A highly sensitive gold nanoparticle bioprobe based electrochemical immunosensor using screen printed graphene biochip. RSC Adv..

[35] Nagatani N., Inoue Y., Araki A., Ushijima H., Hattori G., Sakurai Y., Ogidou Y., Saito M., Tamiya E. (2016). Rapid sensing of antioxidant capacity based on electrochemiluminescence induced by electrochemically generated reactive oxygen species. Electrochim. Acta.

[36] Fujigaki H., Inaba M., Osawa M., Moriyama S., Takahashi Y., Suzuki T., Yamase K., Yoshida Y., Yagura Y., Oyamada T. (2021). Comparative Analysis of Antigen-Specific Anti-SARS-CoV-2 Antibody Isotypes in COVID-19 Patients. J. Immunol..

[37] Divya V.C., Sathasivasubramanian S. (2014). Estimation of serum and salivary immunoglobulin G and immunoglobulin A in oral pre-cancer: A study in oral submucous fibrosis and oral lichen planus. J. Nat. Sci. Biol. Med..

[38] Dave P.K., Rojas-Cessa R., Dong Z., Umpaichitra V. (2020). Survey of Saliva Components and Virus Sensors for Prevention of COVID-19 and Infectious Diseases. Biosensors.

