# Supplementary material for: Point-of-Care Diagnostic Biosensors to Monitor Anti-SARS-CoV-2 Neutralizing IgG/sIgA Antibodies and Antioxidant Activity in Saliva
Source: Biosensors (Basel). 2023 Jan 20;13(2):167. doi: 10.3390/bios13020167 (PMC9953869; doi:10.3390/bios13020167)
Supplement: Supplementary file 1 [file biosensors-13-00167-s001.zip › biosensors-2153061-supplementary.pdf]

Supplementary Materials

Figures S1

After dilution of the saliva samples, known concentrations of IgG were added, and the current responses were examined. High background currents and poor calibrability were observed in the absence of dilution, whereas 10- and 100-fold dilution provided good calibrability.

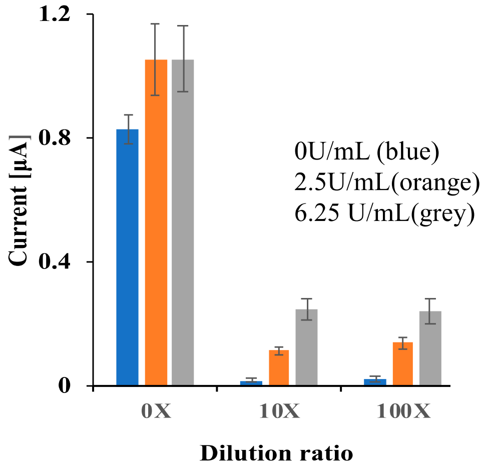

Figure S1. Optimization of the dilution rate for saliva sample.

Figure S2

All 19 saliva samples shown in Figure 5 were tested using immunochromatography test strips (Perio screen, Sunstar, Japan) to determine whether the saliva samples were contaminated with blood. No blood was detected above the detection limit (left panel). The test paper was confirmed to have a detection sensitivity of approximately 3 μg/mL (right).

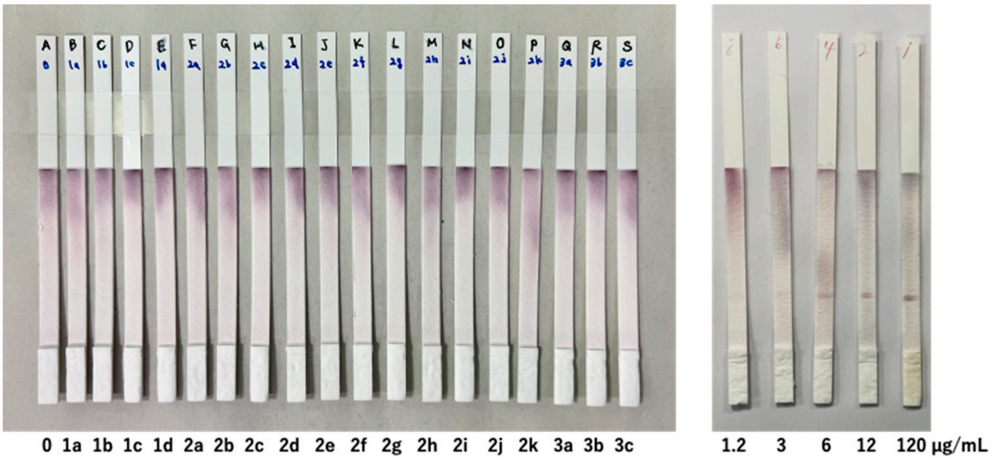

Figure S2. Preliminary check of blood contamination on saliva samples.

Figure S3: Optimization of primary antibody concentration using the electrochemical impedance spectroscopy (EIS). X-axis shows anti-IgA antibody concentration dropped onto the working electrode. Y-axis shows the electron transfer resistance (Rct) which indicate the amount of the antibody adsorbed on the electrode surface. In this case, Rct was saturated over 50 ug/mL of antibody.

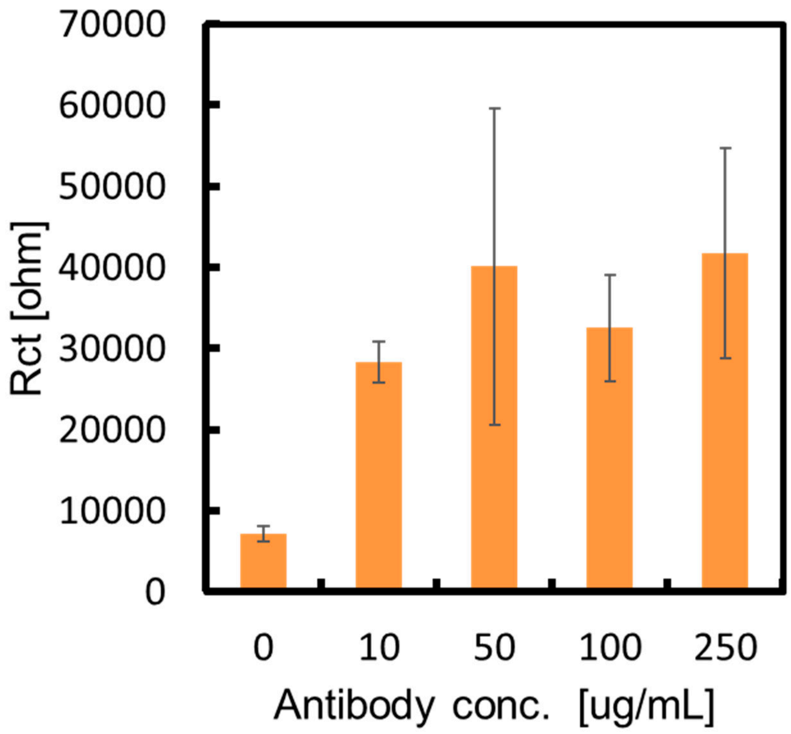

Figure S3. Optimization of primary antibody concentration using the electrochemical impedance spectroscopy (EIS).
